# The Role of 3D-Printed Custom-Made Vertebral Body Implants in the Treatment of Spinal Tumors: A Systematic Review

**DOI:** 10.3390/life12040489

**Published:** 2022-03-28

**Authors:** Roberta Costanzo, Gianluca Ferini, Lara Brunasso, Lapo Bonosi, Massimiliano Porzio, Umberto Emanuele Benigno, Sofia Musso, Rosa Maria Gerardi, Giuseppe Roberto Giammalva, Federica Paolini, Paolo Palmisciano, Giuseppe Emmanuele Umana, Carmelo Lucio Sturiale, Rina Di Bonaventura, Domenico Gerardo Iacopino, Rosario Maugeri

**Affiliations:** 1 Neurosurgical Clinic, AOUP “Paolo Giaccone”, Post Graduate Residency Program in Neurologic Surgery, Department of Biomedicine Neurosciences and Advanced Diagnostics, School of Medicine, University of Palermo, 90127 Palermo, Italy; brunassolara@gmail.com (L.B.); lapo.bonosi@gmail.com (L.B.); massimiliano.porzio1@gmail.com (M.P.); umberto.emanuele.benigno@gmail.com (U.E.B.); sofiamusso.sm@gmail.com (S.M.); rosamariagerardimd@gmail.com (R.M.G.); robertogiammalva@live.it (G.R.G.); federicapaolini94@gmail.com (F.P.); gerardo.iacopino@gmail.com (D.G.I.); rosario.maugeri1977@gmail.com (R.M.); 2 Department of Radiation Oncology, REM Radioterapia s.r.l., 95125 Catania, Italy; gianluca.ferini@grupposamed.com; 3 Trauma Center, Gamma Knife Center, Department of Neurosurgery, Cannizzaro Hospital, 95100 Catania, Italy; paolo.palmisciano94@gmail.com (P.P.); umana.nch@gmail.com (G.E.U.); 4 Fondazione Policlinico Universitario A. Gemelli IRCCS, Università Cattolica del Sacro Cuore, 00100 Rome, Italy; cropcircle.2000@virgilio.it (C.L.S.); rina.di.bonaventura@hotmail.it (R.D.B.)

**Keywords:** 3D print, custom-made implant, vertebral prothesis, spinal tumor

## Abstract

In spinal surgery, 3D prothesis represents a useful instrument for spinal reconstruction after the removal of spinal tumors that require an “en bloc” resection. This represents a complex and demanding procedure, aiming to restore spinal length, alignment and weight-bearing capacity and to provide immediate stability. Thus, in this systematic review the authors searched the literature to investigate and discuss the advantages and limitations of using 3D-printed custom-made vertebral bodies in the treatment of spinal tumors. A systematic literature review was conducted following the PRISMA (Preferred Reporting Items for Systematic Reviews and Meta-Analyses) statement, with no limits in terms of date of publication. The collected studies were exported to Mendeley. The articles were selected according to the following inclusion criteria: availability of full articles, full articles in English, studies regarding the implant of 3D custom-made prothesis after total or partial vertebral resection, studies regarding patients with a histologically confirmed diagnosis of primary spinal tumor or solitary bone metastasis; studies evaluating the implant of 3d custom-made prothesis in the cervical, thoracic, and lumbar spine. Nineteen published studies were included in this literature review, and include a total of 87 patients, 49 males (56.3%) and 38 females (43.7%). The main tumoral location and primary tumor diagnosis were evaluated. The 3D custom-made prothesis represents a feasible tool after tumor en-bloc resection in spinal reconstruction. This procedure is still evolving, and long-term follow-ups are mandatory to assess its safeness and usefulness.

## 1. Introduction

Custom-made implants, anatomical models, molds used to create prothesis or surgical guides have been widely employed since the discovery of 3D printing and its advantages. Thanks to its various applicational fields, three-dimensional printing is gaining an essential role in the medical community [1,2].

In spinal surgery, 3D prothesis represents a useful instrument for spinal reconstruction after the removal of spinal tumors that require an “en bloc” resection (i.e., benign aggressive bone tumors, malignant primary bone tumors, and highly selected cases of spinal metastases). This represents a complex and demanding procedure, aiming to restore spinal length, alignment and weight-bearing capacity and to provide immediate stability [3,4,5,6,7]. 

In the last few years, the use of 3D printing custom-made implants is becoming more considerable in spinal oncology, due to various paramount advantages: 3D printing provides customized implants according to the patient’s specific anatomy and needs (i.e., shape, width, and length of the endplates) and proportioned to the expected extent of resection, ensuring spinal homeostasis and higher success rates thanks to proper osseointegration [8,9]. 

Thus, in this systematic review, the authors searched the literature to investigate and discuss the advantages and limitations of using 3D-printed custom-made vertebral bodies in the treatment of spinal tumors.

## 2. Materials and Methods

### 2.1. Study Design

**Figure 1:**
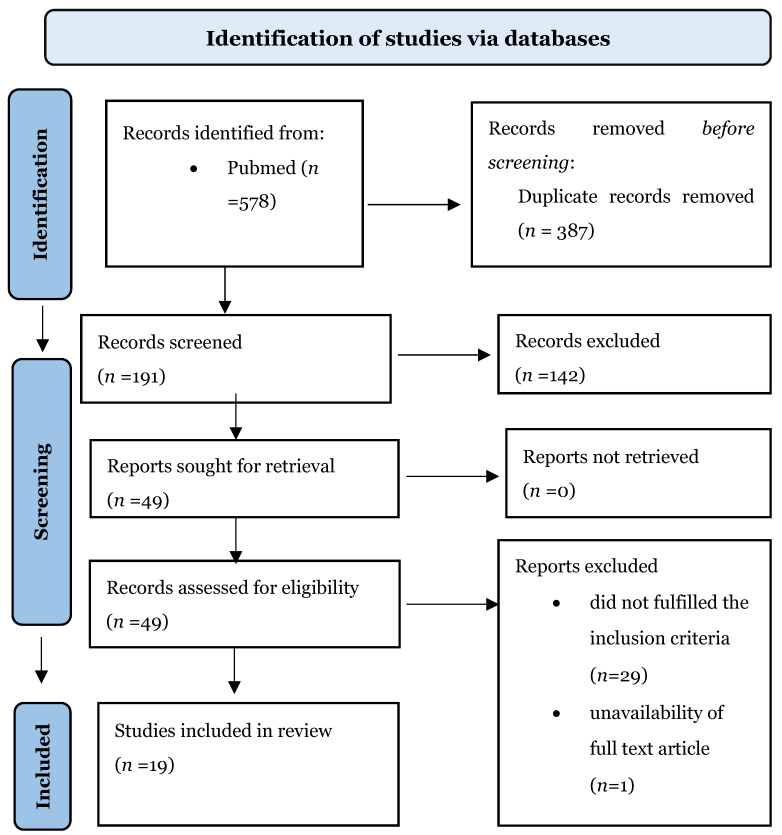
PRISMA 2020 flow diagram.

A systematic literature review was conducted following the PRISMA (Preferred Reporting Items for Systematic Reviews and Meta-Analyses) statement, with no limits in terms of date of publication. The following medical subject headings (MeSH) and free text terms were combined: “3D custom made”, “3D prothesis”, “3D printed implant”, “3D printing”, “additive manufacturing” AND “spinal tumor”, “tumor spine surgery”, “vertebrectomy”, “corpectomy”, “spondylectomy”, “bioengineering”, “titanium”, and “3D printed custom made vertebra”. The collected studies were exported to Mendeley. Duplicates were removed. Details of the search strategy are shown in Figure 1.

### 2.2. Eligibility Criteria

Studies regarding 3D custom-made prothesis after total or partial vertebral resection, studies regarding patients with histologically confirmed diagnosis of primary spinal tumor or solitary bone metastasis; and studies evaluating the implant of 3D custom-made prothesis in the cervical, thoracic, and lumbar spine were included. Studies were excluded if they were: full articles in languages other than English, studies reporting the use of 3D custom-made prothesis only for modeling, studies regarding patients with non-oncological spinal diseases, or studies with implantation of 3D custom-made prothesis in the sacrum. Patients’ demographics are shown in Table 1.

### 2.3. Data Extraction

The available data included authors, year, study design, vertebral localization, histological diagnosis of tumor, presenting symptoms, surgical treatment, type of prothesis implanted, blood loss during surgery, and post-operative course.

## 3. Results

A total of 578 published studies were identified through PubMed, Google Scholar, Scopus databases and additional reference list searches. After performing a first screening by title and abstract reading, 387 papers were excluded because of overlapping results. After a detailed examination of these studies, 142 more papers were excluded (see details in *Eligibility Criteria*).

Hence, 19 published studies were included in this literature review, and include a total of 87 patients, 49 males (56.3%) and 38 females (43.7%). The reported median age was 43.14 ± 15.99 years (range 12–72). Tumors were located at the cervical (28%), cervico-thoracic (1%), thoracic (44%), thoraco-lumbar (5%), lumbar (19%), and lumbo-sacral (1%) spine, and were not applicable for 2% of them. The primary tumor diagnoses were giant cell tumor in 15(17.2%), metastasis 13 (14.9%), chordoma 12 (13.8%), chondrosarcoma 10 (11.5%), osteosarcoma 6 (6.9%), Ewing sarcoma 4 (4.6%), fibrous tumor 3 (3.4%), hemangioma 3 (3.4%), osteoblastoma 3 (3.4%), malignant peripheral nerve sheath tumors 3 (3.4%), undifferentiated pleomorphic sarcoma (UPS) 2 (2.3%), myeloma 1 (1.1%), meningioma 1 (1.1%), paraganglioma 1 (1.1%), plasmacytoma 1 (1.1%), rhabdomyosarcoma 1 (1.1%), liposarcoma 1 (1.1%), pseudo-myogenic-hemangio-endothelioma 1 (1.1%); the remaining part consists of two oncological patients not included in the study (2.3%) and four non-oncological patients not included in the study (4.6%).

**Figure 2:**
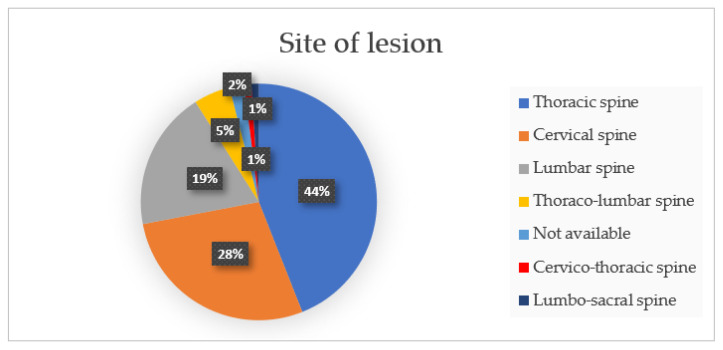
Percentage of spinal segments localization.

**Figure 3:**
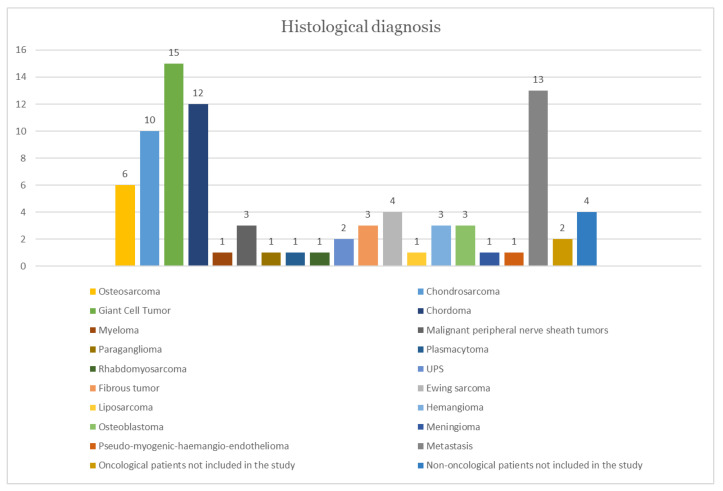
Histogram showing the most common histological diagnoses related to the tumors included in this review.

Complications related to the use of 3D-printed custom-made protheses were not evaluated because they were not fully mentioned in all the articles included in this review. The distribution of tumor localization and the histopathological diagnosis are shown in Figure 2 and Figure 3.

## 4. Discussion

Three-dimensional printing (3DP), also called additive manufacturing, is an emergent technology that is rapidly evolving in medical sectors [27]. It is gaining success in many fields, such as the education of medical students and health-care professionals, preoperative surgical planning, intraoperative applications (patient-specific guides and implants) and, finally, spinal surgery [28].

The peculiarity of 3D-printed technology is its ease of use; CT and MRI can be used by engineers to replace the bony defect through computer-based modelling, using bio-mechanical characteristics that increase the bone match, reducing complication rates [29].

### 4.1. 3D Prothesis Production

**Figure 4:**
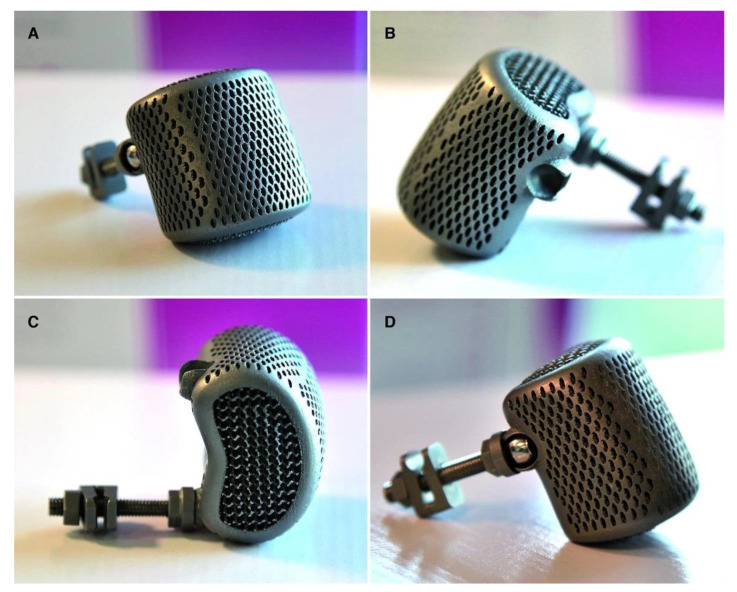
BiomimeTiC titanium cage. Each prosthesis is composed by an innermost three-dimensional lattice structure, mimicking cancellous bone, covered by a fine shell, mimicking cortical bone. Oblique (**A**), lateral (**B**,**D**) and superior views (**C**). Images acquired thanks to Springer permission from the article “Biomimetic 3D-printed custom-made prosthesis for anterior column reconstruction in the thoracolumbar spine: a tailored option following en bloc resection for spinal tumors” Available online: https://link.springer.com/article/10.1007/s00586-018-5708-8 (accessed on 16 March 2022).

The production of 3D models is based on several steps that must be closely followed (Figure 4).

1. Data acquisition: choosing imaging data is one of the most critical steps: a low-resolution image may create a model that does not resemble the real anatomy. The data acquired (DICOM) are processed by 3D software and then segmented and saved as a Standard Tessellation Language (STL) file (the most used format) according to a layer-by-layer building technique.
2. Segmentation: it is an optional step, but in medical fields it is always employed. It is used to select the region of interest and create the surface mesh of the target area (different software can be used to manipulate DICOM data).
3. After the segmentation processes, the voxels extracted are converted into a polygonal model. This process can create artifacts and careful revision and comparison between the region of interest from the processed data and DICOM data are mandatory to guarantee an accurate anatomical prototype.
4. Finally, the STL file can be recognized by the 3D printer software and produced.
5. Post-processing: used to remove excess materials or to smooth prothesis surfaces. Different 3DP materials and techniques can be employed. In spinal surgery, the most used are Selective Laser Sintering (SLS), where several materials as metal alloys or ceramics can be used, and Stereolithography (SLA) [30,31].

### 4.2. Anatomical Modeling

The ability to create 3D anatomical models, in a 1:1 scale, represents a revolution in the surgical field. As a matter of fact, when approaching complex spinal oncological pathologies, in-depth and detailed knowledge of both the anatomy related to the pathology and the exact location of the tumor and its relationships with the surrounding structures is mandatory when the neurosurgeon plans the most suitable surgical treatment, avoiding unexpected problems and reducing the risk of pitfalls [13]. In recent years, thanks to the lower production costs and the spread of 3D printers within a hospital setting, the application of additive manufacturing in preoperative planning has spread enormously. This potential application of 3D printing was also demonstrated in a study by Leary OP et al., where the practical utility of this device in the preoperative planning of a series of nine patients undergoing spinal surgery was evaluated, underlining the usefulness of 3D printing as an adjunct for treating a variety of complex spinal lesions and emphasizing the particular utility of 3D modeling for planning and performing tumor resecting surgeries, preserving healthy structures as much as possible [32].

### 4.3. Medical and Surgical Education

Traditionally, surgeons have learned anatomy from cadaveric specimens, except for those countries where stringent legislation or ethical and religious concerns have forbidden it. In this scenario, the chance to create anatomical models through 3D printing has revolutionized the way students and professionals learn surgical anatomy and surgical procedures [33]. Moreover, 3D printing can be used to print pathologic conditions, such as a vertebral fracture, guarantying a great advantage in terms of learning complex pathologies, as also demonstrated by the study of Li et al [34]. Park et al., indeed, assessed the instructive impact of using a 3D-printed spine model for training residents in pedicle screw freehand instrumentation strategy, emphasizing the advantages and disadvantages of these innovative tools [28,35].

### 4.4. Customization of Surgical Tools

Another interesting field of application of 3D printing is related to the possibility of creating customized surgical instruments, laying the foundations for increasingly personalized surgery based on the individual characteristics of each patient. So, 3D-printing could improve the accuracy and safety of surgical procedures. In this case, 3D prints were exploited to create tools aimed at protecting the dural sac, the spinal cord, or vital vascular structures during the surgical procedure for tumor removal or during osteotomy. For example, a drill guide template with a screw trajectory into the replaced vertebral body has been designed to enhance accuracy, reducing screw deviation and injuries to vital structures, the efficiency, reducing intraoperative time, and the safety, reducing intraoperative radiations [18,36]. In addition, customized instruments have also been created to optimize the positioning of needles for cryotherapy, safeguarding adjacent structures from any thermal damage, to create custom-made screws with customized dimensions and angles and to produce vertebral locking systems based on the specific characteristics of each patient [13].

### 4.5. Surgical Implants and 3D-Prosthesis

Thanks to the creation of custom-made prostheses and customized implants, 3D printing has certainly played a key role. This paved the way for patient-specific medicine and surgery, in particular spinal surgery. The use of 3D patient-specific implants in anatomically challenging cases, such as a tumor that has caused significant structural deformity, appears to be indispensable for improving prognosis [27]. As a matter of fact, in oncological spine surgery the main objective is to perform a total resection of the lesion, trying to preserve structural integrity and spinal stability [36]. Indeed, in primary bone tumors, in order to obtain a free tumor margin of resection and as long as it guarantees a long-term survival, an en-bloc resection with wider margin of excision is mandatory [4,31].

To do this, the prosthetic reconstruction phase has an essential role [37]. Recent studies have shown how an appropriate biomechanical reconstruction can cause fewer complications and reduce the risk of re-intervention [18,36,38]. Indeed, 3D-printed custom-made protheses provide anatomical stability and consequently an improved success rate thanks to the vertebral prothesis designed based on the patient’s characteristics and anatomy.

Moreover, common complications related to prosthetic systems are those related to the process of osseointegration, implant dislocation or hardware failure; with the spread of custom-made 3D printing and with the progressive and constant development of bio-tissue engineering, it is possible to solve, at least in part, these issues [39]. In fact, thanks to the personalized mapping of the vertebral endplates, it is possible, for example, to exploit a wider contact area between the prosthesis and the vertebral interfaces, increasing the stability of the implant and reducing the risk of dislocation or failure [13,40]. Lastly, 3DP technology can produce titanium surfaces with porous scaffolds that allow host bone to grow inside the construct to facilitate integration and promoting the activation of osteogenic cells. Compared to plasma-sprayed porous titanium-coated PEEK and PEEK prothesis that can lead to endplate fracture or implant collapse due to a sharp edge, 3DP decreased the risk of subsistence and damages resulting from radiotherapy [15,29].

This process of osseointegration has been also verified in Wei et al. during the patients’ follow-up, showing, on CT scans, new bone tissue growing around the 3D vertebral prothesis [16].

Furthermore, Girolami et al. evaluated the grade of osseointegration in 3D vertebral body replacement through a histological analysis, showing the presence of bone regeneration and growth in the 3D-printed prothesis, assessing the lower risk of failure, subsidence or migration using these devices. Tong et al [31]. have also enlightened and confirmed that complex spinal cases, such as in patients undergoing vertebral tumor surgeries, could have greater benefits from a 3D-printed prothesis implantation that can ensure proper spine stability, thanks to all the above-mentioned advantages [3,17]. 

As shown in Table 1, the most common site involved was the thoracic tract and the most common tumors were giant cell tumors, metastases and chordomas. Patients’ quality of life (QoL) was severely limited by oncological pain, and an en bloc resection using a 3D custom made prothesis can restore stability, ensuring pain relief and a better QoL. Post-operative complications, whenever reported, are more related to tumor recurrence than 3D vertebral implants, showing a lower rate of subsidence that confirms the usefulness of these protheses [27,28]. Tang et al. showed that subsidence inferior to 2 mm did not affect osseointegration and patient’s outcome [10]. 

Indeed, Girolami et al. underlined how the rate of subsidence was not significant, as long as it did not involve posterior fixation or pain relief [3]. 

Zhuang et al. found that the association of surgical removal of spinal tumors using 3D prothesis vertebra followed by robotic radiosurgery could influence the outcome; indeed, their series has shown no recurrence after radiosurgery. Therefore, this could represent a good compromise to prevent recurrence and, at the same time, guarantee spinal stability [4,16,19,20].

The great opportunity of a custom-made prothesis based on the patient’s peculiar anatomy has simplified reconstructive surgery, especially in those cases where complex anatomy is involved; care and precision must be taken in those districts, such as in the cervical spine, where neurovascular and sensitive anatomic elements such as the larynx, esophagus, vertebral arteries are found. In these districts, 3D printing could show its usefulness both in pre-operative planning and intraoperatively using the custom-made prothesis [41].

### 4.6. Complications

The use of 3D implanted protheses is a promising field that can still display potential pitfalls. Complications in the series revised are commonly related to the surgical procedure, such as CSF leak due to a dural tear, wound infections, transient postoperative neurofunctional deterioration and/or transient minor post-operative dysphagia and dysphonia that fully recovered during the post-operative course [10,15,16].

Nevertheless, it has been reported that the strength of these devices cannot be guaranteed as in conventional implants and that unplanned changes during a surgical procedure can create a mismatch between the prothesis and defect, limiting the capabilities of this device [17,42]. Therefore, the occurrence of subsidence is quite rare but a comparison to conventional techniques is still missing, along with a long-term follow-up.

### 4.7. Limitations

Despite the excellent outcome reported, the use of 3D custom-made protheses is limited by all the unplanned “after printing” complications that can occur and make the prothesis incompatible. To avoid this issue, three-1 mm-different protheses are printed, making costs high and difficult to afford [18].

Indeed, the pre-operative planning, the lack of readily available 3D printers and specialized materials in hospital, and the three different protheses printed are an example of all the limitations that can occur and delay surgical procedures [18].

Another issue enlightened by Chatain et al. is that the FDA approval could take a long time, resulting in a delay to prothesis production: this may represent a critical point, since it affects patients’ quality of life, which has already been influenced by their tumor history. In patients affected by spinal tumor there are no guideline about the choice of which prothesis should be implanted, so each case must be approved by FDA committee [36].

Furthermore, not only approval, but also long manufacturing times have delayed the implementation of this technology in hospitals.

## 5. Conclusions

Three-dimensional custom-made prothesis represents a feasible tool after vertebral tumor en-bloc resection in spinal reconstruction. This procedure is still evolving, and long-term follow-ups are mandatory to assess its safeness and usefulness. Nevertheless, the premises are encouraging, and a wider use of this technology should be considered as a good chance to guarantee an optimal osseointegration with a lower risk of prothesis-related complications.

## Figures and Tables

**Table 1:** TES = Total en bloc spondylectomy; na = not applicable * 4 patients of this study were not included in the present review because the 3D-printed prothesis was not implanted ** one patient of this study was not included in the present review because they were not affected by spinal tumor.

	Authors	Study Design	Level	Tumor	Symptoms	Surgical Treatement	Prothesis	Blood Loss	Further Treatement	Post-Operative Course
1	Girolami M. et al. 2021 [3]	Case report	T12	Primary osteogenic sarcoma	Back pain	En bloc resection and 3D-printed prothesis reconstruction performed by a single posterior approach	Titanium (Ti6Al4 V) printed technology	n/a	-Neo-adjuvant chemotherapy -Adjuvant chemotherapy	-Local recurrence -Re-operation -Surgical site infection -Death 4 months later for disseminate disease
2	Xiaodong Tang et al. 2021 [10]	Retrospective study	-21 thoracic spine -2 thoraco-lumbar spine -4 lumbar spine	-6 chondro-sarcomas -6 giant cell tumors -3 malignant peripheral nerve sheath tumors -2 osteo-sarcomas -2 un-differentiated high-grade pleomorphic sarcomas (UPS) -2 solitary fibrous tumors -1 Ewing’s sarcoma -1 liposarcoma -4 metastatic tumors	n/a	Anterior column 3D-printed prothesis reconstruction after multilevel thoracolumbar TES	Titanium (Ti6Al4 V) printed technology	mean blood loss 4.1 L (range, 0.8–13.3 L)	Chemotherapy, radiation, and targeted therapy in patients with osteosarcoma, chondrosarcoma, malignant peripheral nerve sheath tumor, UPS, and metastatic tumor	Local recurrence in 5 patients At the latest follow-up, in 23 living patients, 19 can walk independently and two can achieve outdoor activities by walking aid. Asymptomatic prosthetic subsidence into adjacent vertebral bodies occurred in 10 patients
3	Xiaodong Tang et al. 2021 [11]	Case report	T1-T5, left upper thoracic cavity and chest wall	Chondrosarcoma	Huge lump involving left shoulder and chest wall; severe radiating pain in the left upper extremity	Multilevel TES Stage 1: anterior “trap door” approach for the exposure of the anterior aspect of the tumor Stage 2: posterior approach for the exposure of the posterior aspect of the tumor Stage 3: lateral approach for tumor removal and 3D-printed prothesis reconstruction	n/a	12.6 L	Preoperative superselective endovascular embolization Adjuvant chemotherapy Targeted therapies	At latest 24-month postoperative follow up, the vertebral prosthesis and internal fixation were intact; there was no tumor local recurrence, and the patient was alive with stable disease.
4	Yuhang Wang et al. 2021 [12]	Case report and literature review	T11-L1	Metastasis from breast cancer	Back pain	One-stage en-bloc spondylectomy of 3-segment tumor lesions via the posterior approach and 3D-printed prothesis reconstruction	Titanium (Ti6Al4 V) printed technology	1.5 L	Radical mastectomy Preoperative selective arterial embolization	At 2 years, no tumor recurrence, no other discomfort and the patient lived well independently
5	Lador R. et al. 2020 [13]	Case series *	-L4-S1 -C3 -T3	-L5 Giant cell tumor -Ewing Sarcoma -Hemangioma	-n/a -n/a -tumor recurrence and local kyphotic deformity	-Complete vertebral resection via midline transperitoneal approach and 3D printed prothesis implantation -complete C3 resection and 3D-printed prothesis implantation	Titanium (Ti6Al4 V) printed technology	n/a	-Percutaneous fixation of L4-S1, and Denosumab -na -Posterior decompressione without instrumentation of T3	n/a
6	Parr W.C.H. et al. 2020 [14]	Case report	C3-C5	Chordoma	Neck and left shoulder pain	Stage 1: posterior tumour resection Stage 2: anterior approach, C3-C5 vertebrectomy, complete macroscopic tumour resection, implantation of the 3D printed PSI	Titanium (Ti6Al4 V) printed technology	n/a	Pre-operative coil embolization of the left vertebral artery proton-beam 135 therapy	At 15 months, satisfactory implant positioning/alignment with no evidence of hardware failure or tumour reoccurrence
7	Hunn S.A.M. et al. 2020 [15]	Case series	C2	-1 metastatic medullary thyroid carcinoma -1 multiple myeloma -1 rheumatoid arthritis **	Neck pain	-oblique anterior cervical approach for tumor resection, implantation of the 3D-printed PSI; posterior fixation C1-C3 -right oblique anterior cervical expposure, resection of C2 and C3, implantation of the 3D-printed PSI; posterior fixation C1-C4	Titanium printed technology	n/a	-primary surgical resection and post-operative radiotherapy	-At 14 months, pain free, neurologically normal and has stable radiological follow up. Her metastatic disease has however progressed in other organ systems. -At 4 months, pain free, stable radiological follow up
8	Wei F. et al. 2020 [16]	Retrospective study	C2 and C2-C3	-1 Ewing’s sarcoma -4 Giant Cell Tumor -1 Paraganglioma -2 Chordoma -1 Hemangioendothelioma	Aggravating pain	Stage 1: posterior midline approach, tumor resection, C2 spondylectomy and posterior fixation Stage 2: anterior high retropharyngeal approach, implantation of the 3D-printed PSI	Titanium (Ti6Al4 V) printed technology	mean blood loss 1.894 L (range, 0.300–6.400 L)	n/a	1 patient died of systemic metastasis and 1 had local tumor recurrence; the other 7 patients were alive and functional in their daily living until the last follow-up without evidence of disease
9	Yang X. et al. 2020 [17]	Case report	C3-T1	Recurrent chordoma	weakness of right upper extremity and burning pain in right forearm	Anteroposterior approach: one-stage intralesional spondylectomy and reconstruction of the cervico-thoracic spine using a customized 3D-printed titanium prosthesis	Titanium (Ti6Al4 V) printed technology	7.5 L	Two surgical treatments for cervical spine chordoma	At 9 months, on local recurrence, no subsidence or dislocation or fractures of the 3D-printed artificial vertebral body
10	Li Y. et al. 2020 [18]	Case report	C1	Solitary plasmacytoma	Neck stiffness and pain	Stage 1: retropharyngeal approach for piecemeal resection of the tumor mass, 3D-printed PSI implantation Stage 2: posterior fixation	Titanium printed technology	1.6 L	Postoperative local radiotherapy	At 12 weeks, 3DP-PSI was in a good position without signs of hardware failure
11	Zhuang H. et al. 2020 [19]	Case series	-5 cervical spine -6 thoracic spine -3 lumbar spine	-1 metastasis of leiomyosarcoma -3 chordomas -2 chondrosarcomas -1 rhabdo-myosarcoma -2 osteosarcomas -1 fibroma -3 osteoblastomas -1 giant cell tumor		-2 subtotal -12 total vertebrectomy anterior (2 patients), posterior (5 patients), or anterior and posterior approaches (7 patients)	Titanium printed technology	Range 0.7–4 L; median 1.1 L	Postoperative radiotherapy	Superior local tumor control was observed in 13 patients, while only 1 patient had recurrence after surgery
12	Peng L. et al. 2020 [20]	Case report	L5-S3	Meningioma	lumbosacral and two legs continuing discomfort and pain and perineal bulge sensation	En bloc resection followed by 3D-printed prosthesis reconstruction	Titanium printed technology	4 L	Preoperative selective arterial embolization	At 4.5 months, the patient could walk short distances with crutches, and the rectum/bladder function was in good condition
13	He S. et al. 2019 [21]	Case report	C2-C7	Chondrosarcoma	right upper extremity weakness and repeated nighttime pain involving the posterior neck	Stage 1: Posterior Approach, Total Laminectomy with Screw-Rod Fixation Stage 2: Anterior-Submandibular Approach, Total Tumor Excision with “Whole-Cervical-Vertebral-Body” 3D-printed microporous titanium prosthesis reconstruction	Titanium printed technology	2.3 L	Postoperative radiotherapy	The patient was able to lead an independent life and go back to work at full capacity by the final follow-up of 14 months
14	Chin B.Z. et al. 2019 [22]	Case report	L2	Recurrence of Giant cell tumor	tenderness in the left lumbar region, radicular pain to the left thigh and knee, and gradual loss of left leg strength	Posteroanterior approach en bloc spondylectomy of L1-L3 with reconstruction using a 3D-printed vertebrae	n/a	2.1 L	Decompression with instrumented fusion of T12-L4	No evidence of GCT recurrence or instrumentation failure at 8- month follow-up
15	Girolami M. et al. 2018 [4]	Prospective observational study	-6 thoracic spine -7 lumbar spine	-1 osteogenic sarcoma -4 chordoma -2 giant cell tumor -1 epithelioid hemangioma -2 metastasis from adenocarcinoma -3 metastasis renal cell carcinoma	Neurologically intact at presentation	In 10 cases, a single vertebral body was resected, while in the remaining 3, the resection involved 2 vertebral bodies. Surgery was performed with a single-posterior approach in 8 of the 9 cases at or above L1, while in the remaining cases (1 at L1 and 4 below L1) an additional anterior approach was necessary	Titanium (Ti6Al4 V) printed technology	n/a	Chemotherapy (1 patient), Denosumab (1 patient)	Subsidence into the adjacent vertebral bodies occurred in all patients; it was clinically irrelevant in (92%). In 1 patient, severity of the subsidence led to revision of the construct. At an average 10-month follow-up (range 2–16), 1 implant was removed due to local recurrence of the disease
16	Choy W.J. et al. 2017 [23]	Case report	T9	Pseudo-myogenic-haemangio-endothelioma	Mid-thoracic pain and a progressive kyphoscoliotic deformity	T9 vertebrectomy from a bilateral costotransversectomy approach, implantation of 3D custom-made prothesis	Titanium printed technology	n/a	Chemotherapy and radiotherapy	The implant was well positioned and had integrated with the adjacent endplates
17	Li X. et al. 2017 [24]	Case report	C2-C4	Metastatic papillary thyroid carcinoma	Neck and upper-extremity pain, dysphagia, and thumb and index finger paresthesia of the right hand	One stage anterior–posterior surgery for radical resection of the metastatic lesion (C2–C4) and thyroid gland, along with insertion of a personalized 3D implant	Titanium (Ti6Al4 V) printed technology	n/a	Radiotherapy	Good cervical vertebrae sequence and position of the 3D printing implant independently engaged in daily activities.
18	Mobbs R. J. et al. 2017 [25]	Case report	C1-C2	-Chordoma -unusual congenital spinal deformity **	Neck and shoulder pain	Posterior Fusion, Oc–C3; Anterior Transoral Approach for en bloc Tumor Resection and 3D Implant Insertion	Titanium printed technology	0.480 L	Postoperative radiotherapy	Normal phonation and swallowing function at his 9-month follow up.
19	Xu N. et al. 2016 [26]	Case report	C2	Ewing Sarcoma	Neck pain paresthesia and clumsiness on both hands	Two- staged intralesional spondylectomy Stage 1: radical excision of the posterior elements of C2 Stage 2: high anterior retropharyngeal approach to remove the remains of C2 and to insert a customized, self-stabilizing artificial vertebral body implant	Titanium printed technology	n/a	Multiagent chemotherapy and local radiotherapy	No subsidence or displacement of the construct, and no local recurrence of the tumor

## Data Availability

Not applicable.
